# Rational Fabrication of Nickel Vanadium Sulfide Encapsulated on Graphene as an Advanced Electrode for High-Performance Supercapacitors

**DOI:** 10.3390/molecules29153642

**Published:** 2024-08-01

**Authors:** Meng Guo, Jia Du, Xueguo Liu, Wentao Liu, Mingjian Zhao, Jianqi Wang, Xuyang Li

**Affiliations:** 1 School of Biological and Chemical Engineering, Nanyang Institute of Technology, Nanyang 473000, China; 2 School of Chemistry and Pharmaceutical Engineering, Nanyang Normal University, Nanyang 473061, China

**Keywords:** nickel vanadium sulfide, graphene, encapsulated, supercapacitors

## Abstract

Supercapacitors (SCs) are widely recognized as competitive power sources for energy storage. The hierarchical structure of nickel vanadium sulfide nanoparticles encapsulated on graphene nanosheets (NVS/G) was fabricated using a cost-effective and scalable solvothermal process. The reaction contents of the composites were explored and optimized. TEM images displayed the nickel vanadium sulfide nanoparticles (NVS NPs) with 20–30 nm average size anchored to graphene nanosheets. The interconnection of graphene nanosheets encapsulating NVS nanoparticles effectively reduces the ion diffusion path between the electrode and electrolyte, thereby enhancing electrochemical performance. The NVS/G composite demonstrated improved electrochemical performance, achieving a maximum of 1437 F g^−1^ specific capacitance at 1 A g^−1^, remarkable rate capability retaining of 1050 F g^−1^ at 20 A g^−1^, and exceptional cycle stability with 91.2% capacitance retention following 10,000 cycles. The NVS/G composite was employed as a cathode, and reduced graphene oxide (rGO) was used as an anode material to assemble a device. Importantly, asymmetric SCs using NVS/G//rGO achieved 74.7 W h kg^−1^ energy density at 0.8 kW kg^−1^ power density, along with outstanding stability with 88.2% capacitance retention following 10,000 cycles. These superior properties of the NVS/G electrode highlight its significant potential in energy storage applications.

## 1. Introduction

In recent years, the challenges of excessive resource consumption and environmental pollution have become more prominent, highlighting the urgent need for new energy materials. Among energy storage devices, supercapacitors (SCs) have garnered a lot of attention because of their high power density, rapid and safe charge–discharge capabilities, and prolonged life cycles [1,2]. However, their low energy density remains a significant barrier, limiting their practical use in advanced electronic devices. The energy storage performance of SCs is closely linked to the characteristics of their electrode materials. Therefore, developing low-cost electrode materials with long life cycles and high energy storage capacities is a crucial challenge for the commercialization of SCs.

Currently, many materials, including nitrides, transition metal oxides, and transition metal chalcogenides (TMCs), have attracted widespread attention [3,4,5]. These electrode materials, with pseudocapacitive and battery-type capacitive characteristics, can achieve battery-level energy density while keeping the same power density as double-layer capacitors [6,7]. In particular, bimetallic sulfides like cobalt copper sulfide (Co_2_CuS_4_) [8] and nickel cobalt sulfide (NiCo_2_S_4_) [9] not only improve the cycle stability of electrodes but also enhance their capacitance. The two metal ions in bimetallic active materials exhibit rich redox reaction characteristics and have low activation energy, facilitating easier electron transfer between ions [10]. This results in higher electrical conductivity compared to monometallic materials. Bae et al. [11] utilized a rapid and environmentally friendly method to fabricate hierarchical nickel cobalt sulfide@C electrode materials, demonstrating excellent control over the material structure. They achieved 334.7 and 242.0 mAh g^−1^ specific capacitances at 2 and 40 A g^−1^. Nickel vanadium sulfide (NVS) has recently emerged as a promising bimetallic sulfide due to its impressive theoretical capacitance and excellent redox performance in energy storage applications [12,13]. Vanadium shows stable oxidation states (ranging from +2 to +5), with higher states capable of storing charge at positive potentials [14,15]. The combined effect between nickel and vanadium contributes to NVS’s exceptional specific capacity. At 1 A g^−1^, Chai et al. [16] synthesized hierarchical porous NVS nanospheres, which displayed an excellent capacitance of 697.4 C g^−1^ and exceptional cycling stability, retaining 86.1% capacity after 3000 cycles at 10 A g^−1^. However, the inherent issue with simple transition metal nanostructures used as electroactive materials is their tendency to agglomerate during SC operation [17]. This agglomeration reduces the number of active sites and the specific surface area (SSA) of the material, thereby hindering improvements in SC capacitance.

To address these challenges, it is essential to develop a conductive substrate that combines with NVS. This substrate could effectively prevent the aggregation and degradation of active NVS while enhancing electron transport kinetics for long-term cycling performance [18]. Among various conductive supports, graphene has garnered significant interest as an ideal substrate due to its ultrahigh surface area and excellent cycling stability [19,20]. This study uses reduced graphene oxide (rGO) as a support to improve the electrode material’s performance by coupling it with active NVS nanoparticles (NPs). The large SSA of rGO minimizes NVS aggregation, thereby promoting electron transfer kinetics and enhancing capacitive performance.

Herein, the hierarchical structure of nickel vanadium sulfide nanoparticles anchored on graphene (NVS/G) was constructed via a one-step solvothermal method. The graphene framework contains uniformly dispersed NVS NPs in this composite. This structure effectively facilitates electron transport, prevents self-agglomeration of active NVS NPs, and enhances cycling stability. The highly conductive graphene network also accelerates electrolyte ion diffusion to access active NVS sites and promotes rapid electron transport, thereby improving electrochemical performance. At 1 A g^−1^, the NVS/G composite showed a high specific capacitance of 1437 F g^−1^, great rate capability, and good cycle stability when used as a positive electrode material. Asymmetric supercapacitors (ASCs) were fabricated using the NVS/G composite and rGO as the positive and negative electrode materials, respectively. These ASCs had a high energy density at 56.5 W h kg^−1^ and power density at 0.823 kW kg^−1^.

## 2. Results and Discussion

### 2.1. Morphology and Structural Analysis

**Scheme 1:**
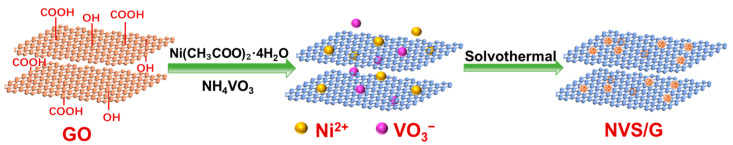
Illustration of the synthesis process of NVS/G composite.

**Figure 1:**
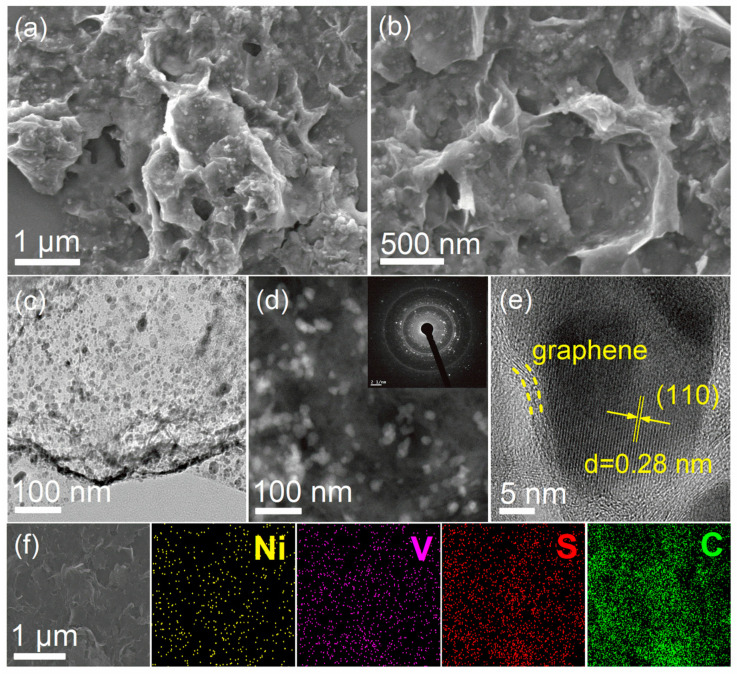
(**a**,**b**) SEM images, (**c**) TEM image, (**d**) STEM image, (**e**) HR-TEM image, and (**f**) EDS mapping of NVS/G-2 composite.

The NVS/G composite was synthesized by a simple and cost-effective solvothermal route illustrated in Scheme 1. The morphology of the hierarchical NVS/G-2 composite was analyzed using FE-SEM, as illustrated in Figure 1a,b. The images reveal that the ultrafine NVS NPs were uniformly encapsulated by rGO nanosheets. This encapsulation effectively prevented aggregation and degradation during charge–discharge cycles, leading to enhanced electrochemical performance [21]. Further detailed morphology was determined using TEM and HR-TEM. Figure 1c shows a TEM image of the composite, displaying NVS NPs with 20–30 nm average size anchored by rGO sheets. This observation was confirmed by the STEM image in Figure 1d, which demonstrated the synergistic effect between active NVS and graphene sheets. The selected area electron diffraction (SAED) pattern in the inset of Figure 1d indicates the crystal nature of the NVS/G-2 composite [22]. The HR-TEM image in Figure 1e demonstrates that NVS NPs were encapsulated by rGO sheets, indicating the growth restraint of NVS NPs by the rGO substrate. This hierarchical structure, derived from the hydroxyl groups of GO and the metal precursors used in the synthesis, reduced NVS NP aggregation and enhanced cycling stability during long-term consecutive cycles [23]. Moreover, the interconnected rGO nanosheet-encapsulated NVS NPs reduced the ion transport pathway length between the electrolyte and electrode, thereby enhancing electrochemical performance [24,25]. Interestingly, the HR-TEM image shows lattice fringes with an approximately 0.32 nm interplanar spacing, which correspond to the (110) plane of NiV_2_S_4_ (JCPDS 36-1132) [26]. The composition and elemental distribution of the NVS/G-2 composite were further analyzed using STEM-EDS mapping, as depicted in Figure 1f. This analysis revealed well-incorporated nickel, vanadium, and sulfur elements within the rGO sheets. These findings highlight the efficiency of the synthesis method in producing the NVS/G composite without necessitating high-temperature conditions. Furthermore, Figure S1 (see Supplementary Materials) shows the morphology of NVS/G-1, NVS/G-2, NVS/G-3, and NVS/G-4 composites. It was observed that NVS/G-1 had larger NPs on graphene than that NVS/G-2. As the temperature rose to 180 °C and 200 °C, it clearly shows the formation of agglomerated NPs in the NVS/G-3 and NVS/G-4 composites. Noticeably, we focus on the NVS/G-2 composite because of its most prominent electrochemical property.

**Figure 2:**
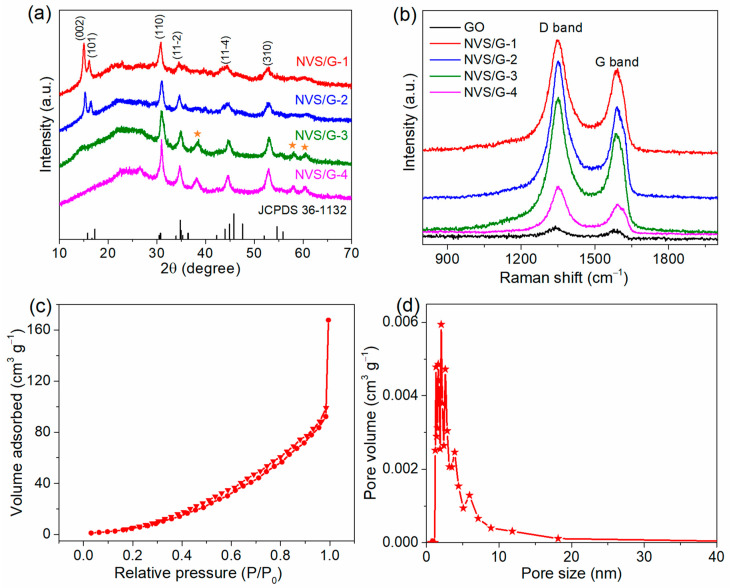
(**a**) XRD pattern; (**b**) Raman spectra of NVS/G-1, NVS/G-2, NVS/G-3, and NVS/G-4 composites; (**c**) N_2_ sorption isotherms; and (**d**) BJH pore size distribution of NVS/G-2 composite.

To further analyze the crystal structure, the XRD patterns of the NVS/G-1, NVS/G-2, NVS/G-3, and NVS/G-4 composites are shown in Figure 2a. A broad diffraction peak around ∼25.8° was observed in all composites, attributed to the (002) plane of graphite from the rGO sheets. The diffraction patterns of the samples aligned well with the standard card data of NiV_2_S_4_ (JCPDS 36-1132) [26], showing strong peaks at 16.0°, 17.4°, 30.9°, 35.0°, 45.1°, and 54.9° corresponding to the (002), (101), (110), (11-2), (11-4), and (310) crystal planes of NiV_2_S_4_, respectively. However, the peaks at 16.0° and 17.4° in NVS/G-3 and NVS/G-4 composites are weak, indicating lower crystallinity. Besides, NVS/G-3 and NVS/G-4 composites showed extra peaks at 38.6°, 58.5° and 60.9° indicated by star, which is ascribed to the Ni_9_S_8_ (JCPDS 22-1193) [27]. This suggests that when the reaction temperature increases to 180 °C and 200 °C, two phases appear in the samples. The crystallite sizes of NVS/G-1, NVS/G-2, NVS/G-3, and NVS/G-4 composites were calculated using the Debye Scherer formula [28]:

D=kλβcos⁡θ

where *D* is crystallite size, *k* is a dimensionless shape factor, *λ* is the wavelength, *β* is the full width at half maximum (FWHM), and *θ* is the Bragg angle. The calculated crystallite sizes of NVS/G-1, NVS/G-2, NVS/G-3, and NVS/G-4 composites were 28.6 nm, 22.6 nm, 23.8 nm, and 38.9 nm, respectively. This result is consistent with the SEM and TEM results. NVS/G-1 showed a larger crystallite size compared to NVS/G-2. As the temperature increased to 180 °C and 200 °C, the crystallite size increased, indicating more agglomerations in the NVS/G-3 and NVS/G-4 composites.

Raman spectroscopy is another effective technique for investigating the structural properties of carbon-based materials. Figure 2b illustrates the Raman spectra of NVS/G-1, NVS/G-2, NVS/G-3, and NVS/G-4 composites. The D and G bands at approximately 1352 and 1591 cm^−1^, respectively, are characteristic Raman shifts indicating the presence of graphitic structures [29]. The intensity ratios between the D and G bands (I_D_/I_G_) for NVS/G-1, NVS/G-2, NVS/G-3, and NVS/G-4 composites were 1.25, 1.31, 1.36, and 1.57, which were higher than those of GO (1.02), suggesting a reduction in graphene oxide during the solvothermal process in all samples [29]. NVS/G-2 showed the most promising electrochemical performance. It is well-established that active electrode materials with a high SSA can considerably improve the electrochemical performance of SCs [30]. The SSA of the NVS/G-2 composite was examined using N₂ sorption isotherm analysis, as depicted in Figure 2c. The composite revealed a type-IV isotherm, indicative of a mesoporous structure [31]. The Barret–Joyner–Halenda (BJH) pore size distribution (Figure 2d) indicated pore diameters around 2.5–3 nm, primarily due to the internal spaces within the active NVS NPs, confirming the mesoporous structure [32]. The NVS/G-2 composite displayed a significantly high SSA of 211 m^2^/g. The high SSA, combined with its unique mesoporous structure, is beneficial for shortening ion diffusion pathways and improving the composite’s electrochemical performance.

**Figure 3:**
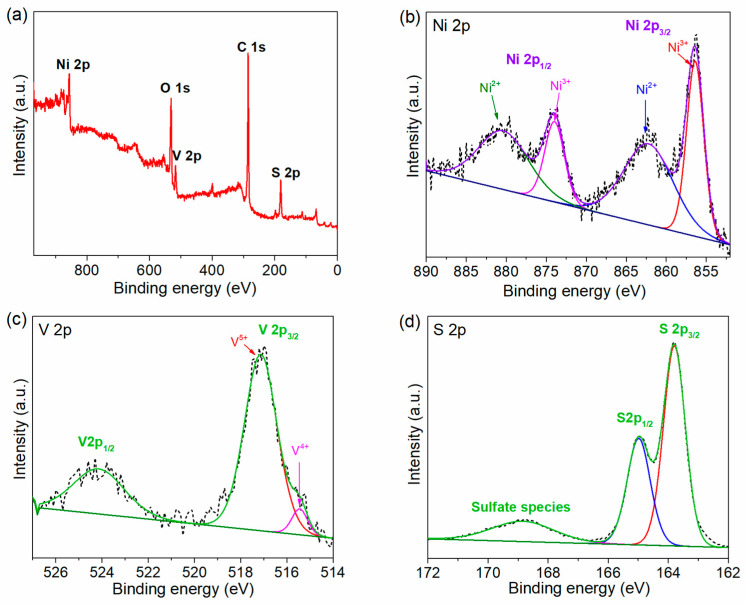
(**a**) XPS survey, (**b**) Ni 2p spectrum, (**c**) V 2p spectrum, and (**d**) S 2p spectrum of NVS/G-2 composite.

To examine the valence states and elemental composition of the hierarchical NVS/G-2 composite, XPS analysis was conducted, as depicted in Figure 3. The existence of Ni 2p, V 2p, S 1s, C 1s, and O 1s was confirmed by the survey spectra in Figure 3a, indicating that the solvothermal reaction successfully formed the NVS/G-2 composite. The high-resolution Ni 2p spectrum (Figure 3b) shows main peaks at approximately 856.5 and 874.2 eV, corresponding to Ni 2p_3/2_ and Ni 2p_1/2_, respectively. These peaks were further divided, indicating the presence of both Ni^2+^ and Ni^3+^ states within the composite [16]. The deconvoluted V 2p spectrum (Figure 3c) reveals two peaks located at 517.1 eV and 524.2 eV for the V 2p_3/2_ and V 2p_1/2_. The V 2p_3/2_ spectra were divided into two peaks, which corresponded to the oxidation states of V^4+^ and V^5+^, respectively [33]. This demonstrates the multiple oxidation states in the NVS/G-2 composite that enhance redox reactions and improve the electrochemical characteristics of SCs. The S 2p spectra in Figure 3d exhibit peaks at 163.7 and 165.0 eV, corresponding to S 2p_3/2_ and S 2p_1/2_, respectively, along with an additional peak at 168.8 eV linked to sulfate species [34]. The chemical composition of as-synthesized NVS/G-2 examined from XPS is depicted in Table S1. The loading of active NVS in the NVS/G-2 composite was 14.68%. The C 1s peaks for C=C, C-O, and C=O bonds are shown in Figure S2 at 284.6, 285.5, and 288.0 eV, respectively [35]. These results collectively confirm the successful formation of the hierarchical NVS/G-2 composite.

### 2.2. Electrochemical Analysis

**Figure 4:**
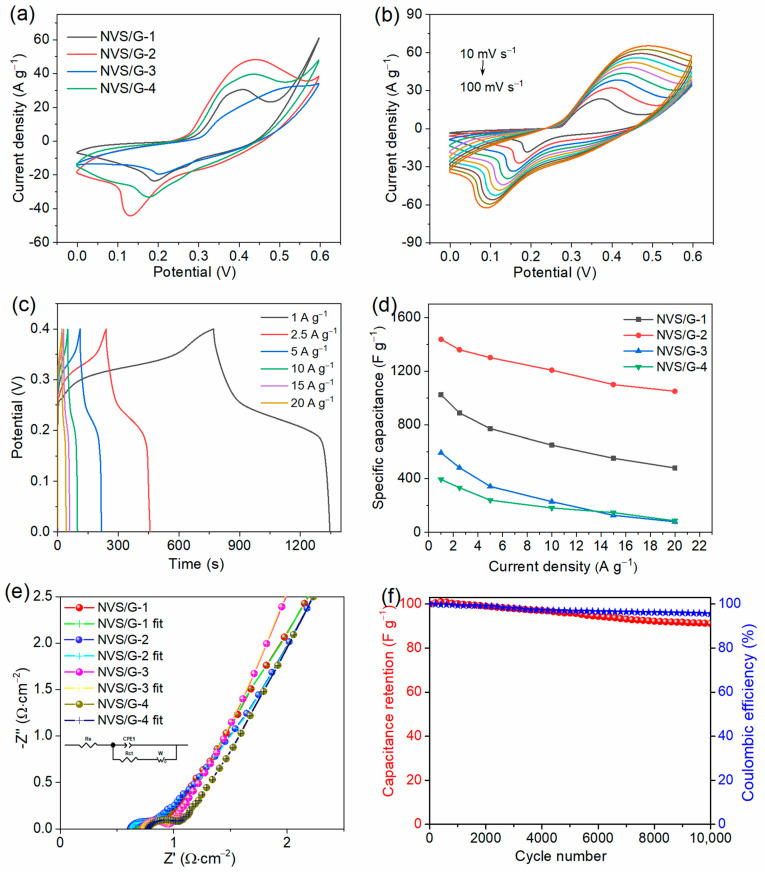
(**a**) CV curves of NVS/G-1, NVS/G-2, NVS/G-3, and NVS/G-4 at 50 mV s^−1^; (**b**) CVs at various sweep rates (10–100 mV s^−1^) of the NVS/G-2 composite; (**c**) GCD curves at different current densities of the NVS/G-2 composite; (**d**) specific capacitance; (**e**) Nyquist plots of NVS/G-1, NVS/G-2, NVS/G-3, and NVS/G-4 composites; and (**f**) cyclic stabilities and coulombic efficiency for 10,000 cycles of NVS/G-2 composite.

To examine the feasibility of the NVS/G-2 composite as a cathode in SCs, its electrochemical performance was investigated with 3 M KOH serving as the electrolyte in a three-electrode setup. Figure 4a displays the CV curves for NVS/G-1, NVS/G-2, NVS/G-3, and NVS/G-4 composites at a 50 mV s^−1^ scan rate. All CV curves revealed prominent redox peaks, indicating battery-type electrode characteristics [36]. The CV integral area for the NVS/G-2 composite was the largest among the samples, signifying its superior electrochemical performance. Figure 4b presents the CV curves of the NVS/G-2 composite at scan rates ranging from 10 to 100 mV s^−1^. These curves consistently demonstrated clear redox peaks, confirming battery-type behavior during the charge–discharge process. Moreover, the peak current increased with higher scan rates, indicating rapid ionic and electronic transport under applied potentials [37]. The electrochemical behavior of the NVS/G-2 composite was further characterized by a GCD test. Figure 4c presents the GCD plots of NVS/G-2 obtained at different current densities between 1 and 20 A g^−1^. The nonlinear curves in Figure 4c were consistent with the CV results and suggest characteristics of battery-type capacitance. Based on the GCD data, specific capacitances were calculated for NVS/G-1, NVS/G-2, NVS/G-3, and NVS/G-4 composites, as depicted in Figure 4d. The NVS/G-2 composite demonstrated outstanding specific capacitances of 1437, 1360, 1301, 1208, 1100, and 1050 F g^−1^ at 1, 2.5, 5, 10, 15, and 20 A g^−1^ current densities, respectively, demonstrating superior performance compared to the other composites. Even at a higher current density of 20 A g^−1^, the NVS/G-2 composite retained 73.1% of its capacitance. These findings are supported by the EIS results shown in Figure 4e. The measured data could be fitted to an equivalent circuit as shown in the inset of Figure 4e. Each plot showed an arc and a straight line in the high- and low-frequency regions, respectively, corresponding to charge transfer resistance (R_ct_) and ion diffusion in the electrolyte [38]. The fitting value of R_s_ and R_ct_ is depicted in Table S2. The NVS/G-2 composite demonstrated a lower R_ct_ value of 0.04 Ω·cm^−2^ compared to NVS/G-1 (0.3 Ω·cm^−2^), NVS/G-3 (0.16 Ω·cm^−2^), and NVS/G-4 (0.32 Ω·cm^−2^). The intercept of the EIS plots at the real axis represents the internal resistance (R_s_) [39]. The NVS/G-2 composite showed the lowest R_s_ at 0.63 Ω·cm^−2^ compared to the NVS/G-1, NVS/G-3, and NVS/G-4 composites. Figure S3 shows the Bode plots of the NVS/G-1, NVS/G-2, NVS/G-3, and NVS/G-4 composites. The relaxation time (τ_0_) was calculated from the characteristic frequency (f_0_) at the phase angle of 45° by τ_0_ = 1/f_0_ [40]. The τ_0_ value of the NVS/G-2 composite was 4.8 s, which was higher than that of the NVS/G-1 (11.1 s), NVS/G-3 (12.5 s), and NVS/G-4 (15.4 s) composites. These results reveal that the NVS/G-2 composite delivered a fast charge transfer and highly efficient ion diffusion, in agreement with EIS results [41]. Figure 4f demonstrates the cycle stability of the NVS/G-2 composite tested over 10,000 charge and discharge cycles at 10 A g^−1^. It retained 91.2% of its capacitance and had 95.2% coulombic efficiency, demonstrating its improved stability. After a long-term charge–discharge test, the SEM image of the NVS/G-2 composite is shown in Figure S4. No separated NVS nanoparticles were observed in the composite, revealing that the NVS nanoparticles were still well encapsulated in graphene sheets. Furthermore, performance of the NVS/G-2 composite was advantageous when compared to the recently reported electrode materials for supercapacitors, as shown in Table 1. The exceptional performance of the hierarchical NVS/G-2 composite can be ascribed to several factors: (i) The presence of multiple valences of vanadium and nickel facilitates rich redox reactions, thereby enhancing capacitive performance. (ii) The synergy between active NVS NPs and thin graphene sheets in the hierarchical structure reduces the electrolyte diffusion path length. Graphene nanosheets in NVS/G-2 act as efficient electron and ion conductors due to their highly conductive properties, preventing agglomeration and degradation of active NVS sites during prolonged charge–discharge cycles and facilitating effective electrolyte interaction.

**Figure 5:**
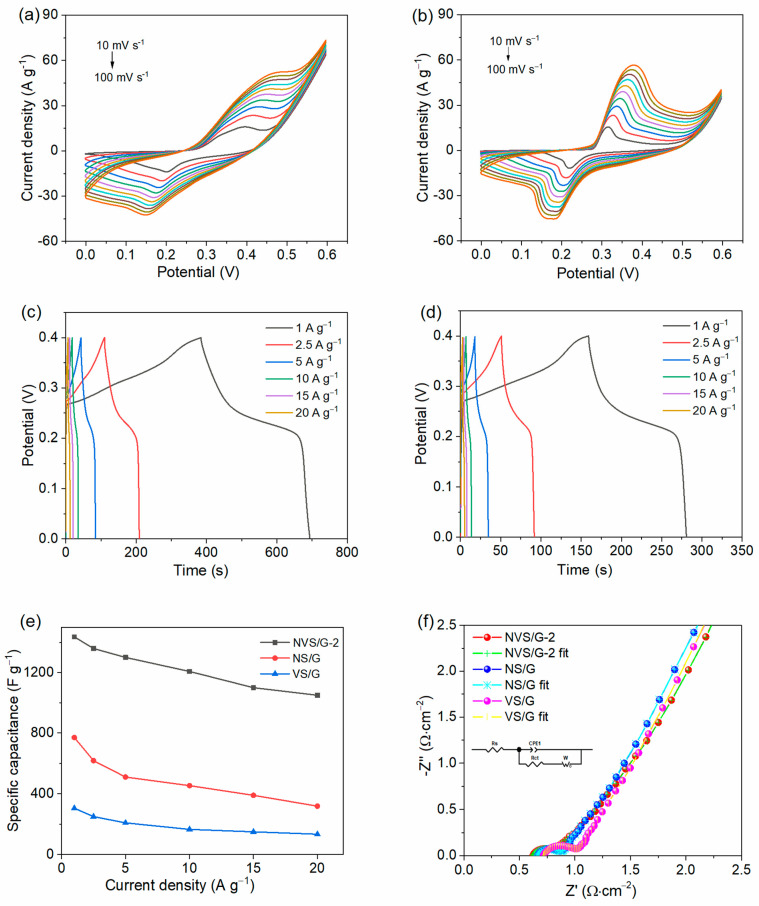
(**a**,**b**) CV curves at various sweep rates; (**c**,**d**) GCD curves at various current densities of NS/G and VS/G composites; (**e**) specific capacitance; and (**f**) Nyquist plots of NVS/G-2, NS/G, and VS/G composites.

To compare with single-metal sulfides, the electrochemical performance of nickel sulfide on graphene and vanadium sulfide on graphene composites was evaluated, as depicted in Figure 5. Figure 5a,b show CV curves at different scan rates for NS/G and VS/G. Both composites demonstrated strong redox peaks, indicating capacitive behavior governed by battery-type characteristics. According to the GCD curves in Figure 5c,d, the specific capacitances of NS/G and VS/G electrodes were calculated as 770 and 305 F g⁻^1^, respectively, at 1 A g⁻^1^. These values were significantly less than the NVS/G-2 composite, as illustrated in Figure 5e. EIS analysis in Figure 5f and the fitting data in Table S3 revealed that the R_ct_ of NVS/G-2 was measured at 0.04 Ω·cm^−2^, which was less than that of NS/G (0.13 Ω·cm^−2^) and VS/G (0.32 Ω·cm^−2^) electrodes. This indicates faster electron transport kinetics in NVS/G-2 due to synergistic interactions between nickel and vanadium ions, facilitating multiple redox reactions [51,52]. Furthermore, the R_s_ values for NS/G, VS/G, and NVS/G-2 electrodes were 0.67, 0.71, and 0.63 Ω·cm^−2^, respectively. The NVS/G-2 electrode displayed lower values for both R_ct_ and R_s_, highlighting the enhanced electrochemical behavior facilitated by mixed oxidation states from nickel and vanadium ions [53].

**Figure 6:**
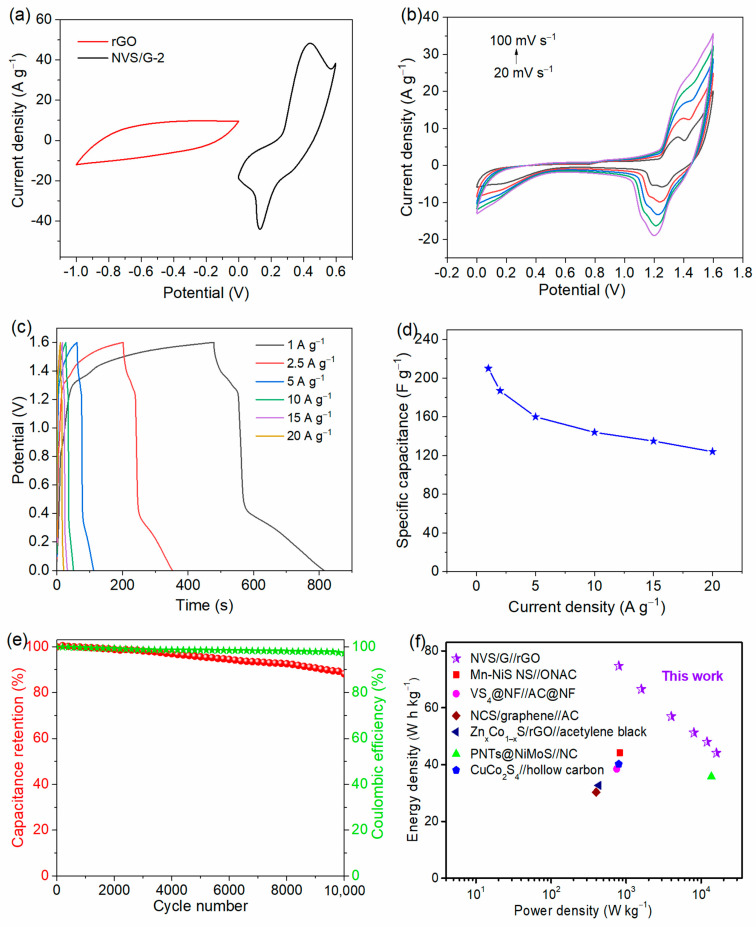
(**a**) CV curves of NVS/G-2 and rGO electrodes at 50 mV s^−1^, (**b**) CVs at various sweep rates (20–100 mV s^−1^), (**c**) GCD curves at various current densities (1–20 A g^−1^), (**d**) specific capacitance vs. current density, (**e**) cycling stabilities for 10,000 cycles, and (**f**) Ragone plot of the NVS/G-2//rGO ASC device. Data of recently reported ASC devices are shown for comparison.

To assess the practical energy storage capabilities of the NVS/G-2 composite in SCs, an ASC was constructed with NVS/G-2 and rGO as the positive and negative electrodes, respectively. Figure 6a shows that the rGO electrode showed typical characteristics of electrical double-layer capacitance within the voltage range between −1.0 and 0 V. In contrast, the NVS/G-2 electrode displayed excellent battery-like behavior within the positive potential range between 0 and 0.6 V. The operational potential window of the ASC was expanded because of the overpotential brought on by the carbon-based negative electrode’s reversible hydrogen electrosorption [54]. To determine the optimal potential window for the assembled NVS/G-2//rGO ASC device, CV and GCD tests were performed across various potential ranges, as illustrated in Figure S5. Extending the potential to 1.8 V resulted in distinct polarization, indicating the onset of the oxygen evolution reaction (OER) [55]. In contrast, the potential range of 0 to 1.6 V remained stable for the ASC device. Consequently, CV plots were conducted at different scan rates within this stable potential window, as depicted in Figure 6b. All CV plots showed redox behavior with consistent curve shapes, indicating efficient electron transfer and rapid charge–discharge characteristics of the ASCs. Figure 6c displays the GCD curves for different current densities. The assembled ASC device’s specific capacitance was calculated based on these curves, and the findings are presented in Figure 6d. At 1 A g^−1^, the specific capacitance reached 210 F g^−1^. Even at higher current densities, such as 20 A g^−1^, the specific capacitance remained stable at 124 F g^−1^, demonstrating an excellent capacitive performance. Moreover, the cycling life of the NVS/G-2//rGO ASC device was tested over 10,000 consecutive GCD cycles at 10 A g⁻^1^, as shown in Figure 6e. The ASC device retained 88.2% of its capacitance after 10,000 cycles, indicating outstanding long-term cycling performance. Upon 10,000 cycles of charge and discharge, the ASC device’s coulombic efficiency remained at 97.2%, demonstrating improved cycling stability and good reversibility. Various energy densities were compared using the Ragone plot to evaluate their electrochemical performance. Figure 6f displays the energy density against power density, or Ragone plot, for NVS/G-2//rGO asymmetric capacitors along with other recent reports. At an 800 W kg⁻^1^ power density, the NVS/G-2//rGO ASCs achieved a 74.7 W h kg⁻^1^ energy density, demonstrating superior performance compared to other previously reported ASC devices, such as Mn-NiS NS//ONAC (44.2 W h kg^−1^ at 825 W kg^−1^) [56]^,^ VS_4_@NF//AC@NF (38.5 W h kg^−1^ at 750 W kg^−1^) [57], NCS/graphene//AC (30.3 W h kg^−1^ at 400 W kg^−1^) [58], Zn_x_Co_1–x_S/rGO//acetylene black (32.7 W h kg^−1^ at 432 W kg^−1^) [59], PNTs@NiMoS//NC (35.8 W h kg^−1^ at 13,719 W kg^−1^) [60], CuCo_2_S_4_//hollow carbon (40.2 W h kg^−1^ at 799 W kg^−1^) [8]. The NVS/G-2//rGO ASC showed superior power and energy densities, making it a feasible choice for useful energy storage applications, including portable electronics and hybrid electric vehicles. The obtained electrode material was prepared by a simple and facile preparation method that achieved excellent performance for supercapacitors. It is an efficient and low-cost manufacturing process. In this synthesis procedure, a relatively lower temperature was employed, which reduced the waste of energy and resources. It is expected to be helpful with reducing unit cost and improving economic efficiency in mass production.

## 3. Materials and Methods

### 3.1. Materials

Nickel acetate tetrahydrate (Ni(CH₃COO)₂·4H_2_O), thioacetamide, ammonium metavanadate (NH_4_VO_3_), potassium hydroxide (KOH), Poly(vinylidene fluoride) (PVDF), N-methyl-2-pyrrolidinone, ethylene glycol (EG), and graphene oxide (GO) powder were obtained from Aladdin Ltd., Shanghai, China. All chemicals were utilized without any further purification.

### 3.2. Preparation of the NVS/G Composite

The synthesis procedure was conducted in a typical process [61], as follows: GO powder (40 mg) was dissolved in 20 mL of EG and sonicated for 40 min. Separately, nickel acetate tetrahydrate (0.25 mmol), ammonium metavanadate (0.5 mmol), and thioacetamide (2.5 mmol) were dissolved in 10 mL of EG. After 30 min of mixing and stirring, the solutions were put in a 50 mL Teflon-lined stainless-steel autoclave and heated to 160 °C for 12 h. After being cooled to room temperature, the final product was washed repeatedly with distilled water and anhydrous ethanol. Following drying at 70 °C, the sample was designated as NVS/G-2. To optimize conditions, similar composites (NVS/G-1, NVS/G-3, NVS/G-4) were prepared at 140 °C, 180 °C, and 200 °C, respectively, using the same method. For comparison, single-metal nickel sulfide/graphene (NS/G) and vanadium sulfide/graphene (VS/G) were prepared using the same procedure. The NS/G composite was synthesized without ammonium metavanadate, and VS/G was synthesized without nickel acetate tetrahydrate.

### 3.3. Characterization of Materials

The synthesized materials were characterized using various analytical techniques. Morphological observations were conducted using a JSM-7900F field emission scanning electron microscope (FE-SEM) from Hitachi (Tokyo, Japan) and a Tecnai G2 F20/F30 transmission electron microscope (TEM). Energy dispersive spectrometry (EDS) was employed to analyze the structural composition and elemental distribution. The crystal structure patterns were analyzed using powder X-ray diffraction (XRD) with a MininFlex600 instrument from Rigaku, Akishima, Japan. Raman spectra were obtained with a Raman spectrometer (LabRAM HR800, Horiba Jobin Yvon, Longjumeau, France). The pore size distribution and SSA were measured with an SSA-4300 apparatus. X-ray photoelectron spectroscopy (XPS) analysis of chemical composition was performed with a Thermo ESCALAB 250XI instrument from the Waltham, MA, USA.

### 3.4. Electrochemical Analysis

The specimens’ electrochemical characteristics were assessed using a conventional three-electrode setup. A slurry comprising carbon black, PVDF, and the sample in a 1:1:8 ratio was prepared in N-methyl-2-pyrrolidinone. After ultrasonication, the mixture was coated onto nickel foam measuring 1 cm × 1 cm, dried in an oven, and used as the working electrode. Ag/AgCl and Platinum foils were employed as reference and counter electrodes, respectively. The mass loading of the obtained NVS/G composite was 4 mg. The cyclic voltammetry (CV) measurement, galvanostatic charge–discharge (GCD) test and electrochemical impedance spectroscopy (EIS) were performed using a 3 M KOH solution as an electrolyte.

### 3.5. Fabrication of the Asymmetric Device

An ASC device was developed using the NVS/G composite and rGO as the positive and negative electrodes, respectively. The electrolyte used was a 3 M KOH solution. The mass ratio of NVS/G composite to rGO electrode was calculated using the charge balance equation, as described by Equation (1):

(1) $$\frac{m_{+}\;}{m_{-}}=\frac{C_{-}\times {\Delta V}_{-}}{C_{+}\times {\Delta V}_{+}}$$

where *C* denotes the specific capacitance of each electrode, *m* represents the electrode material mass, and Δ*V* signifies the applied potential window of the negative and positive electrodes, respectively. The mass loading of the NVS/G composite was 3 mg, while rGO was loaded at 6.5 mg.

The specific capacitance (*C*_s_) of the electrodes was calculated based on the charge–discharge plots using Equation (2):

(2) $$C=\frac{2I\int Vdt}{mV^{2}\;{|}_{V_{i}}^{V_{f}}}$$

where *m* refers to the active material’s mass loading (g), *I* denotes the discharge current (A), *V* is the applied potential window with respect to the initial and final values of *V_i_* and *V_f_*, and ∫Vdt is the integral current area.

The energy density (*E*, in W h kg^−1^) and power density (*P*, in W kg^−1^) of ASC were accessed using Equations (3) and (4) below:

(3) $$E=\frac{C_{cell}\;{\Delta V}^{2}}{2}$$

(4) $$P=\frac{E}{t_{discharge}}$$

where Δ*V* is the applied voltage range, *C_cell_* stands for the specific capacitance of the ASC device, and *t_discharge_* refers to the discharge time during the GCD test.

## 4. Conclusions

In summary, we fabricated a hierarchical nickel vanadium nanoparticle encapsulated on a graphene composite with a large SSA for a high-property energy storage device through a simple and economical method. The reaction temperature was optimized at 160 °C for optimal morphology and performance. This unique architecture facilitated the diffusion of electrolyte ions in the electrode materials during the charge–discharge process to boost the electrochemical performance of SCs. Compared to the corresponding single metallic NS/G and VS/G composites, the obtained bimetallic NVS/G-2 composite achieved a superior specific capacitance of 1437 F g^−1^ at 1 A g^−1^ with a higher rate capability than the NS/G and VS/G composites. The outstanding performance of the obtained electrode material allows for its practical application in ASC devices. The present study provides a route to fabricate bimetallic sulfide-based electrode materials for energy storage devices.

## Figures and Tables

**Table 1:** Comparison of the electrochemical performance of the reported materials for supercapacitors.

Materials	Specific Capacitance	Electrolyte	Stability	Ref.
C-Zn/Co/S	815 F g^−1^ at 2 mV s^−1^	2 M KOH	83.9% (5000 cycles)	[42]
NiCo_2_S_4_@NC	1021.6 F g^−1^ at 1 A g^−1^	6 M KOH	92.7% (5000 cycles)	[43]
NiV-LDH	465.33 F g^−1^ at 1 A g^−1^	3 M KOH	90.7% (5000 cycles)	[18]
NiAg_2_S	571.2 C/g at 1.4 A g^−1^	1 M KOH	86% (1000 cycles)	[44]
NMS@S-gC	934.2 F g^−1^ at 1 A g^−1^	2 M KOH	89.5% (5000 cycles)	[45]
Ni_4_V_1_-LDH@rGO	1511.1 F g^−1^ at 1 A g^−1^	2 M KOH	85.3% (1000 cycles)	[46]
Ni-VO_2_@C/SO_4_^2−^	355 F g^−1^ at 1 A g^−1^	_	88% (10,000 cycles)	[47]
3D NiV-LDHs	1069 F g^−1^ at 1 A g^−1^	6 M KOH	68% (1500 cycles)	[48]
NiMo_3_S_4_/BP	830 F·g^−1^ at 1 A·g^−1^	0.5 M K_2_SO_4_	86% (6000 cycles)	[49]
CeCoSx-SA/GF	873.3 F·g^−1^ at 1 A·g^−1^	6 M KOH	87.1% (5000 cycles)	[50]
NVS/G	1437 F·g^−1^ at 1 A·g^−1^	3 M KOH	91.2% (10,000 cycles)	This work

## Data Availability

The data that support the finding of this work are available from the corresponding author upon reasonable request.

## Supplementary Materials

The following supporting information can be downloaded at: https://www.mdpi.com/article/10.3390/molecules29153642/s1, Figure S1: SEM images of NVS/G-1, NVS/G-2, NVS/G-3, and NVS/G-4 composites; Figure S2: C 1s spectra of NVS/G-2 composite; Figure S3: Bode plots of NVS/G-1, NVS/G-2, NVS/G-3, and NVS/G-4 composites; Figure S4: SEM image after 10,000 consecutive charge–discharge cycles; Figure S5: CV curves at 50 mV s^−1^ and GCD curves at 5 A g^−1^ of NVS/G-2//rGO ASC device at different potential windows; Table S1: Chemical composition of as-synthesized NVS/G-2 examined from XPS; Table S2: Fitting values of R_s_ and R_ct_ for NVS/G-1, NVS/G-2, NVS/G-3, and NVS/G-4 composites; Table S3: Fitting values of R_s_ and R_ct_ for NVS/G-2, NS/G, and VS/G composites.
