# Supplementary material for: Rational Fabrication of Nickel Vanadium Sulfide Encapsulated on Graphene as an Advanced Electrode for High-Performance Supercapacitors
Source: Molecules. 2024 Aug 1;29(15):3642. doi: 10.3390/molecules29153642 (PMC11313959; doi:10.3390/molecules29153642)
Supplement: Supplementary file 1 [file molecules-29-03642-s001.zip › molecules-3127677-supplementary.pdf]

Rational fabrication of nickel vanadium sulfide encapsulated on graphene as an advanced electrode for high-performance supercapacitors

Meng Guo [1,*], Jia Du [1], Xueguo Liu [1], Wentao Liu [1], Mingjian Zhao [1], Jianqi Wang [1], Xuyang Li [b,*]

[1.] School of Biological and Chemical Engineering, Nanyang Institute of Technology, Nanyang, 473000, China

[2.] School of Chemistry and Pharmaceutical Engineering, Nanyang Normal University, Nanyang, 473061, China

* Correspondence: mengme2007@126.com (M. Guo); eryang1024@163.com (X. Li)

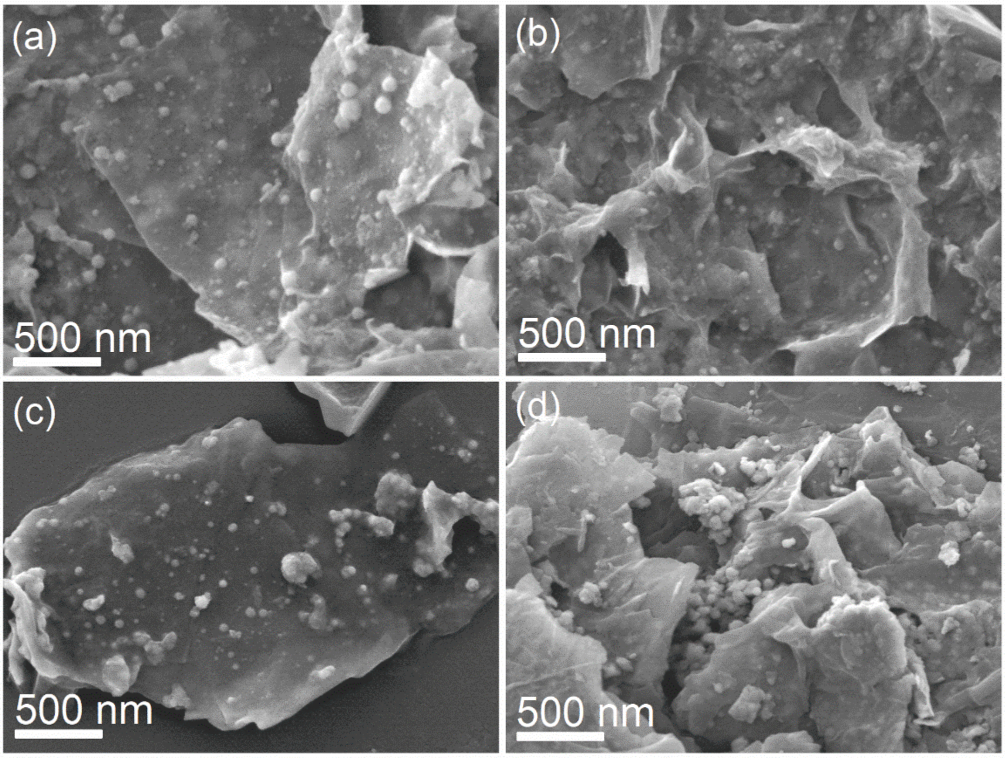

Figure S1. SEM images of NVS/G-1, NVS/G-2, NVS/G-3, and NVS/G-4 composites.

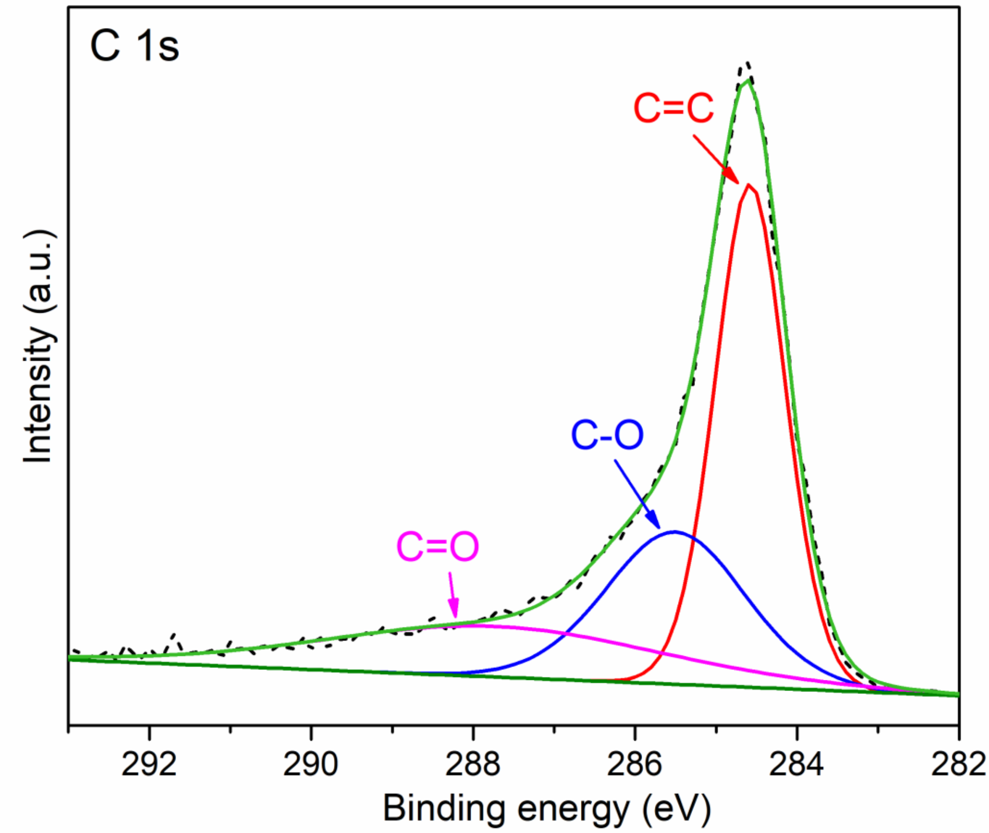

Figure S2. C 1s spectra of NVS/G-2 composite.

Table S1. Chemical composition of as-synthesized NVS/G-2 examined from XPS

Sample	Ni (at. %)	V (at. %)	S (at. %)	C (at. %)	O (at. %)
NVS/G-2	5.3	1.55	7.83	63.81	21.51

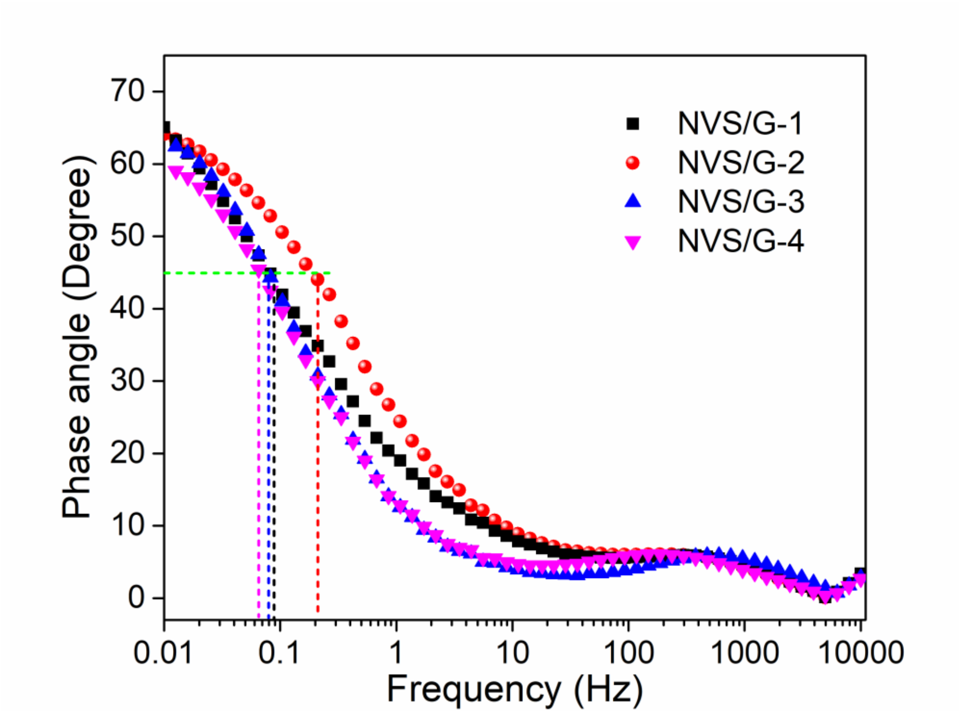

Figure S3. Bode plots of NVS/G-1, NVS/G-2, NVS/G-3, and NVS/G-4 composites.

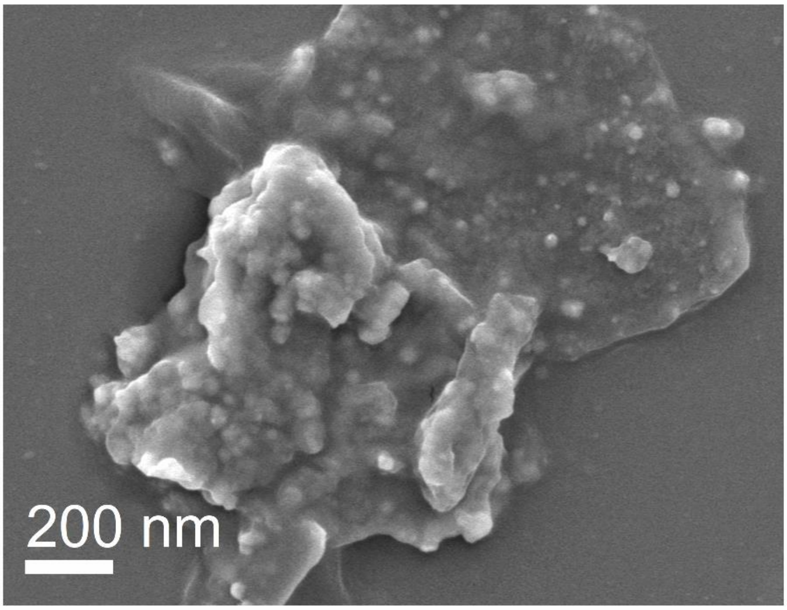

Figure S4. SEM image after 10000 consecutive charge-discharge cycles

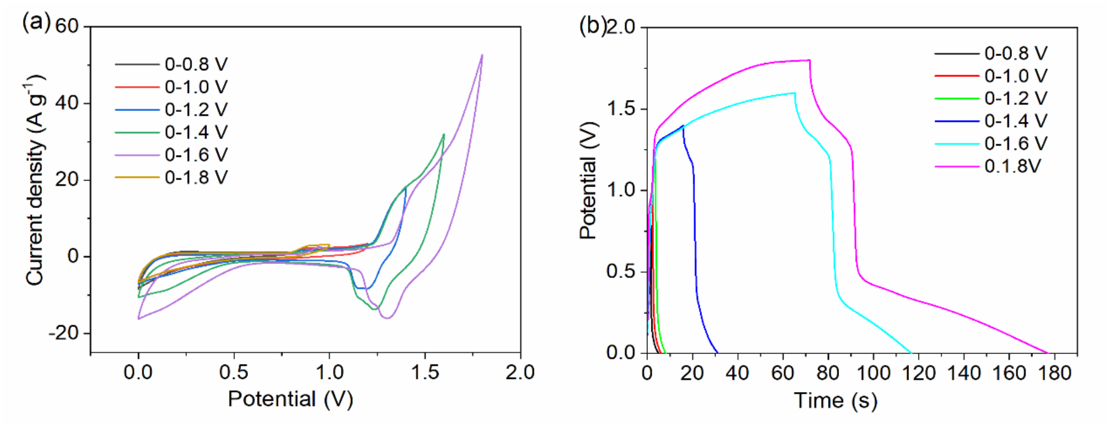

Figure S5. (a) CV curves at 50 mV s^{-1}, and (b) GCD curves at 5 A g^{-1} of NVS/G-2//rGO ASC device at different potential windows.

Table S2 Fitting values of R_s and R_{ct} for NVS/G-1, NVS/G-2, NVS/G-3, and NVS/G-4 composites

Samples	R_s ($\Omega \cdot cm^{-2}$)	R_{ct} ($\Omega \cdot cm^{-2}$)
NVS/G-1	0.69	0.3
NVS/G-2	0.63	0.04
NVS/G-3	0.73	0.16
NVS/G-4	0.77	0.32

Table S3 Fittingvalues of R_s and R_{ct} for NVS/G-2, NS/G, and VS/G composites

Samples	R_s ($\Omega \cdot cm^{-2}$)	R_{ct} ($\Omega \cdot cm^{-2}$)
NVS/G-2	0.63	0.04
NS/G	0.67	0.13
VS/G	0.71	0.32
